# Cordycepin Decreases Ischemia/Reperfusion Injury in Diabetic Hearts *via* Upregulating AMPK/Mfn2-dependent Mitochondrial Fusion

**DOI:** 10.3389/fphar.2021.754005

**Published:** 2021-10-20

**Authors:** Houyou Yu, Xin Hong, Lihua Liu, Yangpeng Wu, Xuemei Xie, Guoxiang Fang, Shaomin Zhi

**Affiliations:** ^1^ Department of Emergency, Xi’an No.3 Hospital, Xi’an, China; ^2^ College of Basic Medicine, Fourth Military Medical University, Xi’an, China

**Keywords:** cordycepin, mitochondrial fusion, diabetes, myocardial ischemia/reperfusion, cardioprotection

## Abstract

Diabetes mellitus is considered to be a major risk factor for cardiovascular disease, the most common cause of death in diabetes. However, therapeutic strategies for myocardial protection in patients with diabetes are still limited. Cordycepin is a traditional Tibetan medicine with a long history of widespread use, and exerts a wide range of anti-tumor, anti-inflammatory, and anti-oxidative effects. In recent years, although the therapeutic potential of cordycepin has attracted the attention of researchers, it remains unknown whether cordycepin plays a protective role in myocardial ischemia/reperfusion (MI/R) injury in diabetic patients. Here, using a diabetic mouse model, we found that cordycepin protected diabetic hearts from MI/R injury by promoting mitochondrial fusion and Mfn2 expression. Our *in vitro* results showed that cordycepin enhanced Mfn2-medicated mitochondrial fusion, improved mitochondrial function, and reduced cardiomyocyte apoptosis in high-glucose/high-fat cultured simulated ischemia/reperfusion cardiomyocytes. Furthermore, we found that knockout of Mfn2 significantly blocked the cardioprotective effects of cordycepin in diabetic mice. Finally, an AMPK-dependent pathway was found to upregulate Mfn2 expression upon cordycepin treatment, indicating that cordycepin protected diabetic hearts *via* AMPK/Mfn2-dependent mitochondrial fusion. Collectively, our study firstly demonstrated that cordycepin could be a potential cardioprotective agent for MI/R injury, and we established a novel mechanism by which upregulated AMPK/Mfn2-dependent mitochondrial fusion contributes to the cardioprotective role of cordycepin.

## Introduction

Cardiovascular disease is the leading cause of death in patients with diabetes (Dillmann, 2019). Previous studies have demonstrated that the incidence rate of cardiovascular disease among patients with diabetes is ∼4-fold higher than that among patients without diabetes (Kannel and McGee, 1979; Mavrogeni et al., 2021). Patients with diabetes are more susceptible to myocardial ischemia/reperfusion (MI/R) injury as evidenced by worse clinical prognosis and higher mortality when compared with patients without diabetes (Whittington et al., 2012; Lejay et al., 2016; Li et al., 2016). Therefore, it is necessary to explore new strategies to prevent MI/R injury in patients with diabetes.

Cordycepin, isolated from Cordyceps mushrooms, is a nucleotide analogue that exerts therapeutic effects as well as nutraceutical potential (Ashraf et al., 2020; Khan and Tania, 2020). Previous studies have reported the anti-inflammatory and anti-oxidative effects of cordycepin. For example, cordycepin exerts its protective effect against lipopolysaccharide-induced acute lung injury and renal ischemia/reperfusion injury (I/R) by regulating inflammation, apoptosis, and oxidative stress (Lei et al., 2018; Han et al., 2020). Nevertheless, the possible effects of cordycepin on the cardiovascular system are rarely discussed, and whether cordycepin exerts a cardioprotective effect following myocardial ischemia/reperfusion (MI/R) injury remains unclear.

Mitochondria are semiautonomous organelles that play a central role in cellular energy metabolism and apoptosis regulation. Mitochondria are highly dynamic and show distinct morphological changes *via* fission and fusion, which play a key role in MI/R processes, such as excessive mitochondrial fission, which is the primary cause of myocardial dysfunction (Milane et al., 2015; Lee and Yoon, 2016; Mui and Zhang, 2021). At the molecular level, mitochondrial dynamics are regulated by crucial mitochondrial membrane proteins. Specifically, dynamin-related protein 1 (Drp1) governs its membrane division, mitofusin 1 (Mfn1), and mitofusin 2 (Mfn2) govern its membrane fusion, and optic atrophy 1 (Opa1) is required for its inner membrane fusion (Whitley et al., 2019). Previous studies have demonstrated that excessive mitochondrial fission is closely related to myocardial injury during ischemia–reperfusion injury in mouse models (Serasinghe and Chipuk, 2017; Mui and Zhang, 2021) a recent study has reported that cordycepin treatment ameliorated aberrant mitochondrial dynamics *in vitro* (Zhang et al., 2021). However, whether cordycepin regulates mitochondrial dynamics following MI/R in diabetes remains unknown.

Adenosine monophosphate-activated protein kinase (AMPK) regulates cellular energy balance and cellular stress in eukaryotic cells. Due to its vital role in controlling energy homeostasis, accumulating studies have indicated that AMPK has attracted widespread interest as a potential therapeutic target for various human diseases (Carling, 2017). In the cardiovascular system, crosstalk between ischemic diseases and AMPK has been studied in the past decades. Numerous studies have found that AMPK activation appears to have a protective effect on cardiomyocytes under MI/R by decreasing apoptosis, improving post-ischemic recovery, and reducing myocardial infarction (MI) (Wu and Zou, 2020). Interestingly, cordycepin has been reported as a pro-drug that activates AMPK by being converted into the AMP analog cordycepin monophosphate (Wu et al., 2014; Wang et al., 2019; Hawley et al., 2020). Moreover, recent studies have demonstrated that AMPK regulates mitochondrial fusion and mitophagy to sustain mitochondrial homeostasis under stress conditions (Toyama et al., 2016; Zhang et al., 2019), indicating the key role of AMPK in mitochondrial dynamics.

Therefore, the aims of the present study were to determine 1) whether cordycepin would provide protection against MI/R injury in diabetes, and if so, 2) to investigate the underlying mechanisms.

## Materials and Methods

### Animal Care and Drug Treatment

All animal experiments were complied with the ARRIVE guidelines and conducted in compliance with the National Institutes of Health Guide for the Care and Use of Laboratory Animals (NIH Publications No. 8023, revised 1978) and were reviewed and approved by the Xi’an No.3 Hospital Committee on Animal Care.

Mfn2^loxp/loxp^ mice with cardiomyocyte-specific deletion of the Mfn2 exon were generated using the Cre-Lox strategy. Briefly, Mfn2^loxp/loxp^ mice were crossed with the Myh6-Cre/ERT2 line. For induction of Cre-mediated deletion of Mfn2, mice were fed 20 mg tamoxifen/kg body weight/day for 7 days. Littermates of Mfn2^loxp/loxp^ mice were used as control animals and were designated as the wild-type (WT) group. Male and female mice were evenly divided into each group. There is no influence of sex on the results of the study.

Four-week-old wild-type C57BL/6 mice were fed a high-fat diet (60% of kcal from fat; Research Diets) for 6 weeks. The mice were injected with streptozotocin (STZ) twice (25 mg/kg i.p.; STZ in 0.05 mol/L sodium citrate, pH 4.5, once daily; Sigma). Blood glucose was measured at 4 weeks after STZ injection, and a diabetic condition was confirmed by a non-fasting blood glucose level of ≥200 mg/dl. Diabetic mice were pre-treated with 10 mg/kg cordycepin (C805132, Macklin, China) orally once a day for a week, as previously described (Park et al., 2014). Other diabetic mice (vehicle groups) received only 0.5% carboxymethylcellulose. The compound C (CC) treatment group was additionally administered CC 20 mg/kg (S7306, Selleck Chemicals) intraperitoneally 1 h before cordycepin.

### Experimental Protocol

The myocardial ischemia/reperfusion (MI/R) model was created by ligation of the left anterior descending artery. Briefly, adult male C57BL/6 mice were anesthetized with pentobarbital sodium (60 mg/kg) *via* intraperitoneal injection, and artificial respiration was established using a ventilator (Model 557040, Harvard Apparatus, Holliston, MA, United States) with a tidal volume 0.2 ml/min and rate of 120 strokes/min). After routine disinfection of the skin, the chest was opened through the left third intercostal space, and the heart was exteriorized through a left thoracic incision by making a slipknot with a 7-0 surgical silk suture to ligate the left anterior descending coronary artery (LAD). Ischemia and reperfusion were monitored and confirmed by electrocardiogram (ECG) observation (success in coronary occlusion was confirmed by immediate ST elevation on electrocardiogram). After 30 min of ligation, the slipknot was released to allow reperfusion for 3 h. The sham-operated control mice underwent the same surgical procedure and the same volume of saline, except for ligation of the heart. The mice were then sacrificed for heart staining or tissue and blood collection. Blood was collected after reperfusion, and the serum was stored at −80°C for further analysis. The hearts were harvested and rinsed with ice-cold phosphate-buffered saline. Ventricular tissue was immediately frozen in nitrogen and stored at −80°C.

### Cell Culture

Primary cardiomyocytes were prepared from neonatal rats (0–2−days old). Besides, the rat cardiac myoblast cell line (H9c2) was purchased from the Cell Bank of Chinese Academy of Science (Shanghai, China). Cardiomyocytes were cultured with normal (5.6 mmol/L glucose, DMEM-low glucose, HyClone) or high-glucose/high-fat (HGHF, 25 mmol/L glucose, and 500 μmol/L sodium palmitate) culture medium for 18 h as previously described (Ji et al., 2017). To establish a simulated ischemia/reperfusion (SI/R) model *in vitro*, neonatal rat cardiomyocytes or H9c2 cardiomyocytes were initially treated with deprivation of both glucose and serum and flushed with 5% CO_2_ and 95% N_2_ in a modular incubator chamber (Billumps-Rothenberg) for 12 h at 37°C. The culture medium was then replaced with fresh oxygenated normal or HGHF culture medium for 6 h of reoxygenation in a normoxic incubator (95% air, 5% CO_2_). 20 μmol/L cordycepin or 0.2% DMSO was added 30 min before HG/HF culturing (Yang et al., 2021).

### Echocardiography Assessment

After 3 h of reperfusion, cardiac function was evaluated by echocardiography, which was conducted with an M-mode Vevo 2100 high-resolution *in vivo* imaging system (VisualSonics, Toronto, ON, Canada) to record the left ventricular systolic and diastolic motion profiles. Before the detection process, the mice were anesthetized by inhalation of 2.5% isoflurane, and anesthesia was maintained with 2% isoflurane at a stable body temperature of approximately 37°C. Left ventricular ejection fraction (LVEF) and left ventricular fractional shortening (LVFS) were measured and analyzed as previously described (Hu et al., 2019).

### Determination of Myocardial Infarct Size

At the end of reperfusion, the myocardial infarct size (INF) was determined using a double-staining technique. The coronary artery was ligated, and Evans blue solution was injected into the left ventricular cavity. The dye was circulated and uniformly distributed except in the region of the heart previously perfused by the occluded coronary artery (ischemic region or area at risk, AAR). The heart was quickly excised, and the atria, right ventricle, and fatty tissues were removed from the heart. After freezing at -20°C, the whole heart was cut into four pieces. Slices were stained with 1% triphenyltetrazolium chloride (TTC) in phosphate buffer (pH 7.4) at 37°C for 10 min. The TTC staining negative areas were infarcted myocardium. Myocardial infarct size was expressed as a percentage of infarct area over total area at risk (infarct area/area-at-risk × 100%).

### Determination of Serum Lactate Dehydrogenase and Creatine Kinase Level

At the end of the reperfusion, blood samples were obtained and immediately centrifuged at 3,000 × *g* for 10 min at 4°C. Serum was collected for subsequent analyses. Serum creatine kinase (CK) and lactate dehydrogenase (LDH) levels were measured spectrophotometrically (DU 640; Beckman Coulter, Brea, CA) in a blinded manner as previously described (Zhao et al., 2020). All measurements were performed in duplicates.

### Transmission Electron Microscopy

Heart tissues were fixed with 2.5% glutaraldehyde in 0.1 M phosphate buffer (pH 7.4, 4°C) for 24 h, rinsed several times with 0.1 M phosphate buffer (pH 7.4), and then post-fixed with 1% osmium tetroxide in deionized water. Morphological changes in mitochondria in slices were viewed and photographed using transmission electron microscopy at 300 kV (JEM-1230, JEOL Ltd., Tokyo, Japan). The images were analyzed by a technician blinded to the treatment, and the length of the mitochondria was measured using ImageJ software. The number of mitochondria was used to analyze mitochondrial morphology, as previously described (Hu et al., 2019). At least 300 mitochondria in a minimum of eight images per mouse were analyzed.

### Western Blotting and Quantitative RT-PCR Analysis

Heart tissue or cardiomyocytes were processed for Western blotting as previously described (Ji et al., 2017). Primary antibodies against the following proteins were used: β-actin (Beijing TDY BIOTEC #TDY051C), Mfn2 (Abcam, #ab56889), cytochrome C (Abcam, #ab133504), cleaved caspase-3 (Cell Signaling Technology, #9664), COX IV (Proteintech, #11242-1-AP), cleaved caspase-9 (Cell signaling technology #20750), phospho-AMPKα (Thr172) (Cell Signaling Technology, #2535), AMPKα antibody (Cell signaling technology, #2532), and Mfn2 polyclonal antibody (Proteintech, #12186-1-AP). A chemiluminescence system (Amersham Bioscience, Buckinghamshire, United Kingdom) was used to analyze the signals of immunoreactive bands. Western blotting results were quantified using ImageJ software (Rawak Software, Inc., Stuttgart, Germany).

Total RNA was extracted from frozen heart tissue using TRIzol reagent and reverse transcribed into cDNA. Quantitative RT-PCR was performed as described previously (Huang et al., 2016). The primer sequences were as follows: Mfn2 forward CTT​GAA​GAC​ACC​CAC​AGG​AAC​A, Mfn2 reverse GGC​CAG​CAC​TTC​GCT​GAT​AC; actin forward GTC​CCT​CAC​CCT​CCC​AAA​AG, and actin reverse GCT​GCC​TCA​ACA​CCT​CAA​CCC.

### Mitotracker Staining

Cardiomyocytes were inoculated into dishes specialized for laser confocal scanning and cultured overnight. The supernatant of the cell culture was discarded the next day and washed twice with PBS. Cardiomyocytes were incubated with preheated 100 mmol/L MitoTracker™ Red CMXRos probe (M7512, Thermo Fisher Scientific, United States) at 37°C for 15 min. The staining solution was discarded, and the dishes were washed with PBS three times (5 min each). Finally, 100 μL of fresh culture medium was added to each well. Images were obtained using a confocal laser-scanning microscope (FluoView FV1000; Olympus, Tokyo, Japan). The effects of cordycepin on mitochondrial morphology in SI/R cardiomyocytes were analyzed.

### Measurement of Oxygen Consumption Rate

Mitochondrial respiration was measured by the oxygen consumption rate (OCR). H9c2 cardiomyocytes at a dentisy of 1.5 × 10^4^ cells per well were cultured into XFp cell culture miniplate (Seahorse Bioscience, North Billerica, MA). Prior to the day of assay, H9c2 cardiomyocytes were pretreated with SI/R induction. On the day of assay, the cultured medium was altered to XF assay medium. After a 1 h incubation in a CO_2_-free chamber, the dissolved O_2_ concentration, in medium immediately surrounding adherent cells, was measured three times at 37°C in an XF24 Extracellular Flux Analyzer (Seahorse Bioscience). The XF Cell Mito Stress Test kit (Seahorse Bioscience) was used to measure the cardiomyocytes which were incubated sequentially under 1 μmol/L of oligomycin, 2 μmol/L of trifluoromethoxy carbonyl cyanide phenylhydrazone (FCCP), 0.5 μmol/L of rotenone and 0.5 μmol/L antimycin A, respectively. The parameters of OCR were measured according to the manufacturer’s protocol by using the Seahorse XFp Extracellular Flux analyzer and software.

### MitoSOX Staining

The fluorescent MitoSOX Red dye (purchased from Molecular Probes, Eugene, OR, United States) was used to measure the dynamic production of superoxide anion (·O_2_^−^), an indicator of reactive oxygen species (ROS) generated in cardiomyocytes within the mitochondria in cardiomyocytes. For this purpose, cardiomyocytes were loaded with 5 μmol/L MitoSOX Red for 10 min kept in dark place, and then washed for 15 min. Neonatal rat cardiomyocytes were excited at 510 nm and emitted light was collected under Fluorescence microscope at 585 nm to obtained the images of cardiomyocytes.

### Measurement of Oxidative DNA Damage in Cardiomyocytes

The cardiac amount of γ-H2A.X, one of the most well-known DNA damage markers of oxidative stress injury, was subjected to Western blot analysis after SI/R induction to determine γ-H2A.X/H2A.X ratio which reflects the DNA damage. Total protein was isolated from neonatal rat cardiomyocytes were processed for Western blotting, as previously described (Ji et al., 2017). Primary antibodies targeted against γ-H2A.X variant histone (H2A.X; cat. no.2577; 1:1,000; Cell Signaling Technology, Inc.) and H2A.X (cat. no.2595; 1:1,000; Cell Signaling Technology, Inc.) were used. Protein expression levels were (semi-)quantified using ImageJ software (Rawak Software, Inc., Stuttgart, Germany). A standard bar graph was generated and used to determine the γ-H2A.X/H2A.X ratio in the samples.

### Mitochondrial ATP Measurements

Cardiomyocytes were washed twice with cold PBS and lysed with lysis buffer containing phosphatase inhibitors for 30 min at 4°C. Samples were centrifuged at 13,000 rpm for 15 min at 4°C, and the supernatant was collected for use in the firefly luciferase-based ATP assay according to the manufacturer’s instructions. The luminescence signal was measured using a luminometer (Tecan Infinite M200 Pro) at 560-nm absorbance. The firefly luciferase-based ATP determination kit (Molecular Probes) was used to measure mitochondrial ATP content. Each assay was performed at least three times (Yan et al., 2019).

### Mitochondrial Glutathione Content

Mitochondrial glutathione (GSH + GSSG) content was determined in lysates from isolated mitochondria using a total glutathione assay kit (Beyotime), according to the manufacturer’s instructions.

### Assessment of Mitochondrial Membrane Potential

Tetramethylrhodamine methyl ester (TMRM; 10 nmol/L; Invitrogen) chemical dye was used for mitochondrial membrane potential measurement according to the manufacturer’s protocol as previously described (Zhao et al., 2020). Before imaging, the cells were rinsed thrice with PBS. A confocal laser scanning microscope (FluoView FV1000; Olympus, Japan) was used for fluorescence image capture and fluorescence intensity analysis, with excitation at 530 nm and emission at 573 nm.

### Cell Apoptosis Assay

According to the manufacturer’s instructions, cardiomyocyte apoptosis was determined by flow cytometry using the Annexin V-FITC Apoptosis Detection Kit (Beyotime, Shanghai, China). In brief, cardiomyocytes were stained with a mixed dye solution of Annexin V-FITC (an indicator of apoptosis) 5 μL (20 μg/L) and propidium iodide (PI) 10 μL (50 μg/L) at 4°C for 30 min incubation. Analysis by flow cytometry (COULTER EPICS XL-MCL; Beckman Coulter, Brea, CA, United States) was performed within 30 min (excitation and emission wavelengths of 485 and 525 nm, respectively).

### Co-Immunoprecipitation

Co-immunoprecipitation (Co-IP) was performed using a Pierce Classic Magnetic IP/Co-IP Kit (Thermo Fisher Scientific, Waltham, MA, United States) according to the manufacturer’s protocol. Briefly, fresh mouse hearts, collected as described above, were homogenized and lysed in cell lysates on ice for 30 min until the lysate was fully clear. Cell lysates were incubated directly with anti-Mfn2 or anti-AMPK immunoprecipitation at 4°C overnight. The antigen/antibody complexes obtained in the previous step were coupled to protein A/G magnetic beads for 1 h at room temperature. After cleaning the unbound protein from the beads several times, the antigen/antibody complex was eluted from the beads with elution buffer. Protein samples containing dithiothreitol were then heated and separated by electrophoresis (SDS-PAGE). After cooling to room temperature and transferring to PVDF membranes, immunoblotting was performed with antibodies to verify the efficiency of immunoprecipitation.

### Statistical Analysis

All values are expressed as mean ± SEM. One-way analysis of variance was performed, followed by the Bonferroni post-hoc test. All statistical analyses were performed using GraphPad Prism version 8.0. (GraphPad Software, La Jolla, CA, United States). Statistical significance was set at *p* < 0.05.

## Result

### Cordycepin Protected Diabetic Hearts From Ischemia/Reperfusion Injury

**FIGURE 1:**
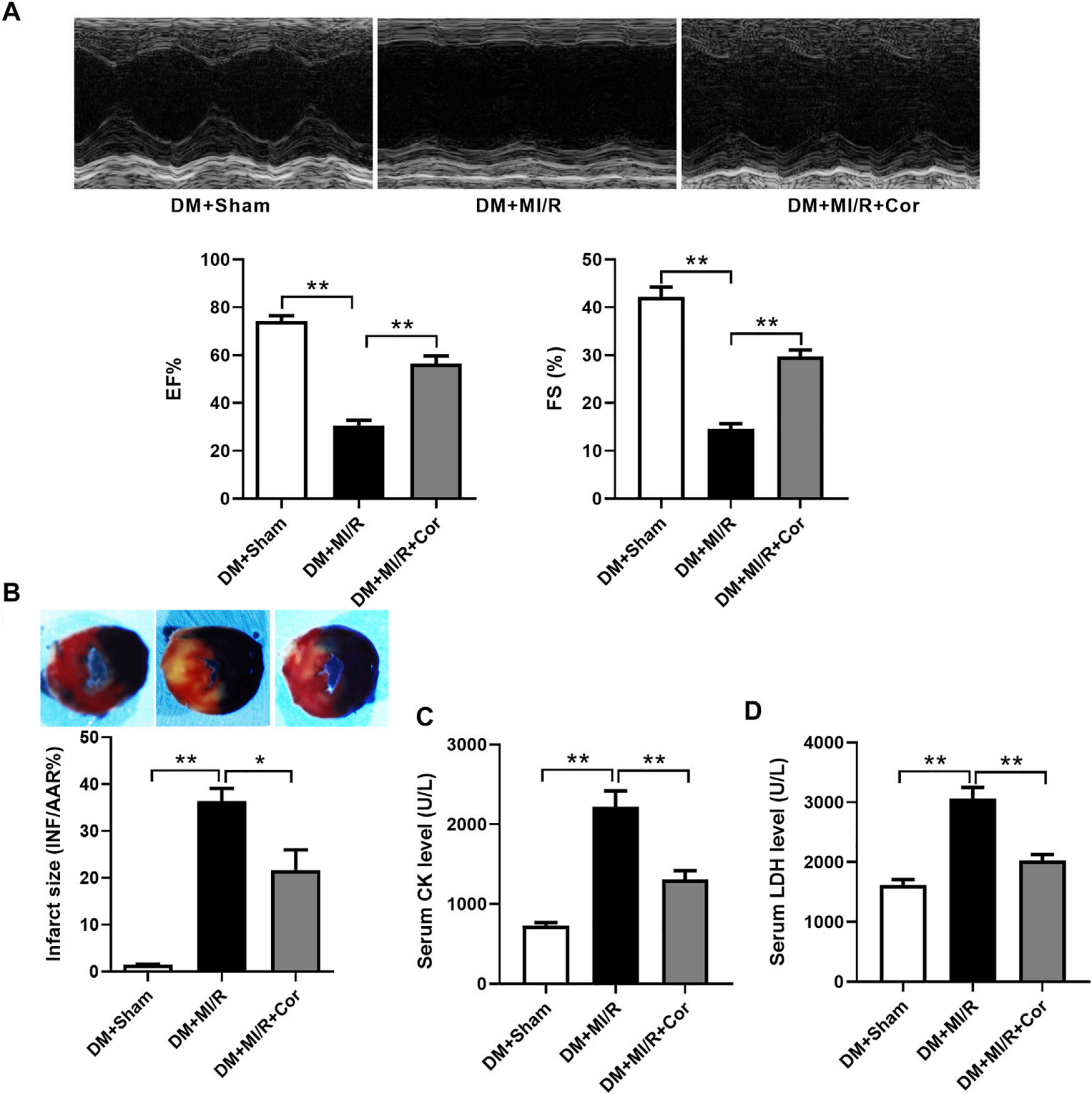
Cordycepin protected diabetic hearts from ischemia/reperfusion injury. **(A)** Top: Representative echocardiograms images from diabetic mice hearts under ischemia/reperfusion injury (with or without cordycepin treatment); Bottom: quantitative assessment of the left ventricular ejection fraction (EF) and left ventricular fractional shortening (FS). **(B)** Top: representative photographs of heart sections. Blue-stained portion indicates nonischemic, normal region; red-stained portion, ischemic/reperfused but not infarcted region; and negative-stained portion, ischemic/reperfused infarcted region; Bottom: myocardial infarct size (INF) analysis in different groups evaluated as percentage of area at risk (AAR). **(C, D)** Effects of Cordycepin on serum creatine kinase (CK) and lactate dehydrogenase (LDH) level after myocardial ischemia/reperfusion injury in different groups. DM, diabetes mellitus; MI/R, myocardial ischemia/reperfusion; Sham, sham-operated control rats; Cor, cordycepin. *p < 0.05, **p < 0.01. *n* = 4–6 mice.

To assess the functional role of cordycepin in diabetic hearts following MI/R, we first examined myocardial function using echocardiography. As shown in Figure 1A, cardiac function was impaired after MI/R treatment, as evidenced by a significant decrease in the left ventricular ejection fraction (EF) and left ventricular fractional shortening (FS) after MI/R injury in DM mice, while the impairment of left ventricular contractile function was mitigated in the cordycepin group compared to the DM + MI/R group (Figure 1A, *p* < 0.01). As described in Figure 1B, myocardial infarct size was found to be elevated by MI/R (*p* < 0.01), and this increase was significantly alleviated by cordycepin treatment (*p* < 0.05). Compared with the sham group, serum creatine kinase (CK) and lactate dehydrogenase (LDH) levels, using to indicate the degree of cardiac injury (Zhao et al., 2020), were significantly increased in MI/R hearts, whereas cordycepin significantly alleviated MI/R injury (Figures 1C,D, *p* < 0.01). These results indicate that cordycepin plays a protective role in diabetic hearts subjected to MI/R treatment.

### Cordycepin Stimulated Mfn2 Expression and Enhanced Mitochondrial Fusion in Ischemic/Reperfused Hearts

**FIGURE 2:**
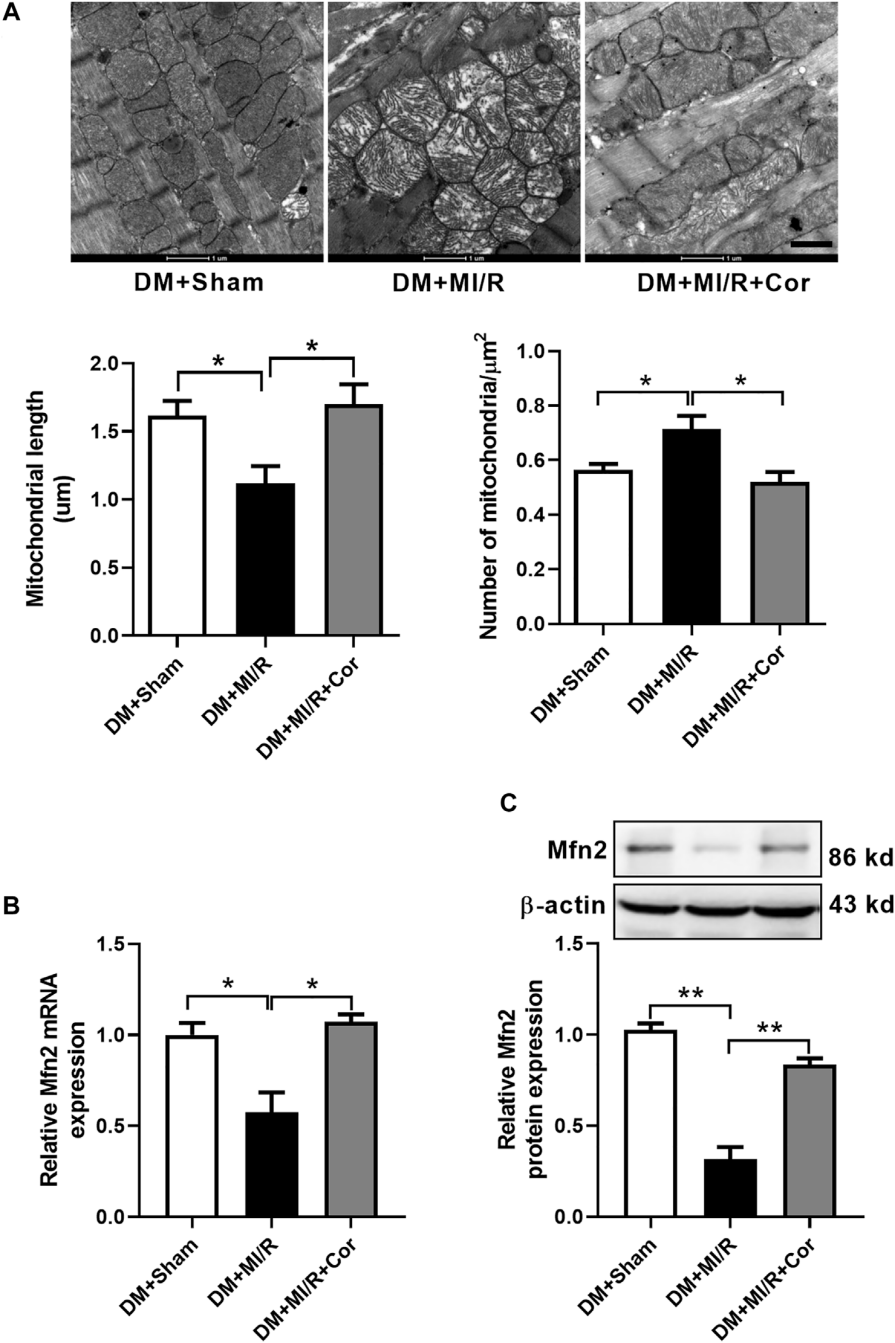
Cordycepin stimulated mitochondrial fusion and enhanced Mfn2 expression in ischemic/reperfused hearts. **(A)** Representative transmission electron microscopic images of mitochondria in heart tissues and quantitative analysis of mitochondria length and the number of mitochondria per um2. **(B)** Quantitative RT-PCR analysis of Mfn2 mRNA expression level in mouse heart. **(C)** Western blotting and quantitative analysis of Mfn2 protein expression levels in heart tissues of mouse. DM, diabetes mellitus; MI/R, myocardial ischemia/reperfusion; Sham, sham-operated control rats; Cor, cordycepin. Scale bar, 1 μm *p < 0.05, **p < 0.01. *n* = 4 mice.

To assess whether cordycepin protect diabetic hearts through regulating mitochondrial dynamics, we examined mitochondrial morphology using Transmission Electron Microscopy (TEM). As shown in Figure 2A, TEM analysis revealed that mitochondria from the hearts of the DM + MI/R group exhibited excessive fragmentation. However, cordycepin efficiently prevented mitochondrial fragmentation, as evidenced by the increased length of mitochondria (*p* < 0.05) and decreased number of mitochondria per μm^2^ (*p* < 0.01) compared with the DM + MI/R group. Moreover, mitochondria in the myocardium from the DM + MI/R group exhibited more obvious swelling, whereas cordycepin treatment significantly reduced the swelling. As illustrated in Figure 2B, the relative mRNA expression of the mitochondrial fusion regulator Mfn2 was markedly upregulated in the DM + MI/R mice treated with cordycepin (*p* < 0.05). Consistently, Western blotting results revealed that the expression level of Mfn2 protein was also upregulated in MI/R hearts following cordycepin treatment (Figure 2C, *p* < 0.05). Therefore, we conclude that cordycepin stimulated Mfn2 expression and enhances mitochondrial fusion in ischemic/reperfused diabetic hearts.

### Cordycepin Enhanced Mfn2-Medicated Mitochondrial Fusion and Improved Mitochondrial Function in HGHF Cultured SI/R Cardiomyocytes

**FIGURE 3:**
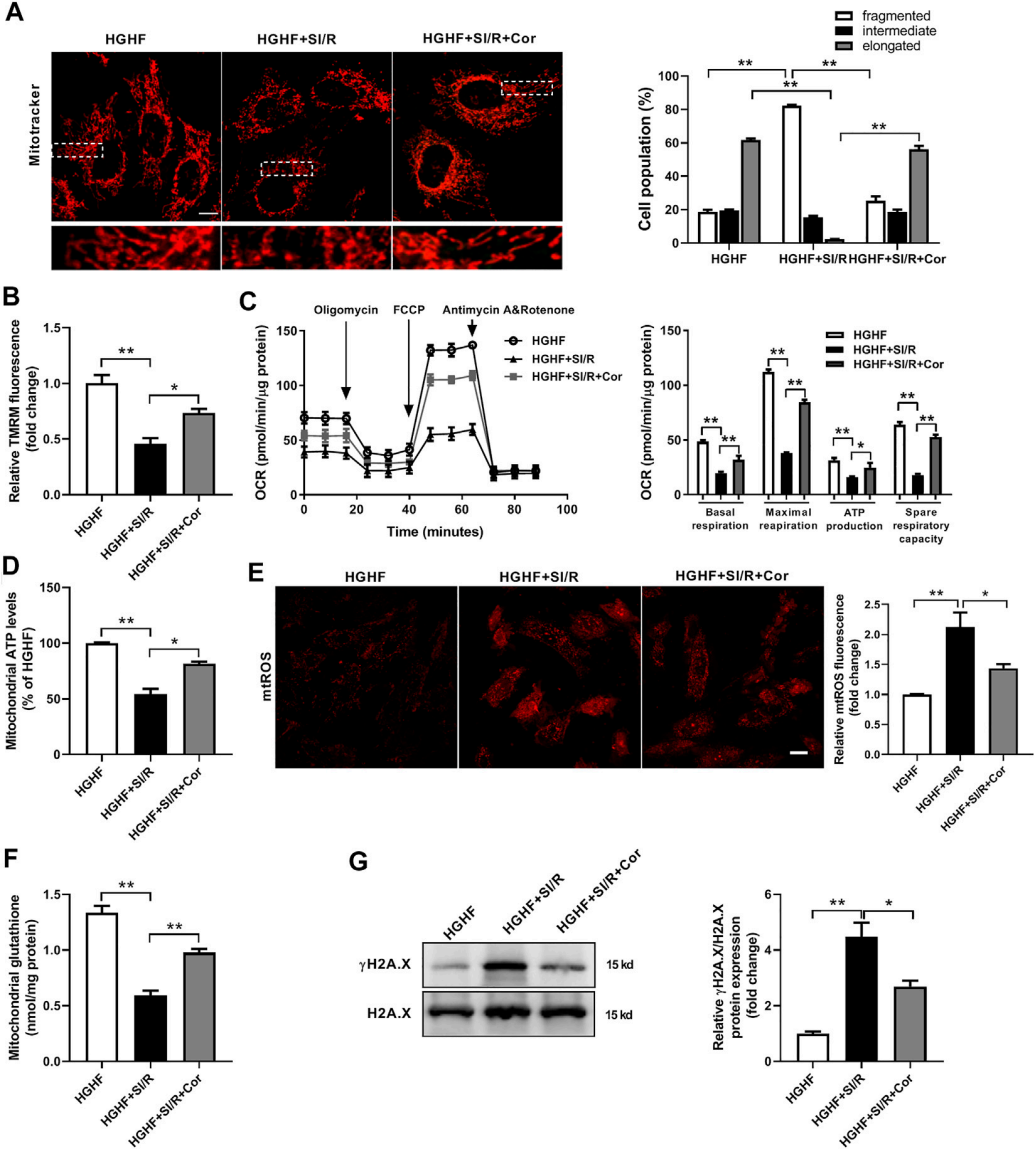
Cordycepin enhanced Mfn2-medicated mitochondrial fusion and improved mitochondrial function in HG/HF cultured SI/R cardiomyocytes. **(A)** Representative images and quantitative analysis of mitochondrial morphology in cardiomyocytes under different treatments. Fifty to 60 cells per sample were counted. Scale bar, 10 μm. **(B)** Quantitative analysis of relative TMRM fluorescence that represented mitochondrial membrane potentials in cardiomyocytes under different treatments. **(C)** Measurement of Oxygen Consumption Rate (OCR) in H9c2 cardiomyocytes in different groups. *n* = 5 wells. **(D)** Measurement of the intracellular ATP levels in different groups. **(E)** Representative images and quantitative analysis of mtROS in cardiomyocytes under different treatments. Scale bar, 20 μm. **(F)** Mitochondrial glutathione content in mitochondria isolated from cardiomyocytes under different treatments. **(G)** Western blotting and quantitative analysis of γH2A.X and H2A.X in cardiomyocytes. *n* = 3 wells. HG/HF, high-glucose/high-fat; SI/R, simulated ischemia/reperfusion; Cor, cordycepin; TMRM, tetramethylrhodamine methyl ester. *p < 0.05, **p < 0.01. *n* = 6 wells.

To further investigate whether cordycepin affects mitochondrial dynamics and function *in vitro*, we used HG/HF-cultured cardiomyocytes isolated from neonatal rats to establish an *in vitro* SI/R model. Consistent with our previous *in vivo* results, HG/HF and SI/R treatment induced excessive mitochondrial fission in cardiomyocytes as shown in Figure 2A, however, cordycepin treatment significantly promoted mitochondrial fusion in HG/HF-cultured SI/R cardiomyocytes (Figure 3A, *p* < 0.05). TMRM was used to determine mitochondrial membrane potential as previously described (Zhao et al., 2020). Our results revealed that SI/R treatment significantly decreased relative TMRM fluorescence in HGHF-cultured cardiomyocytes whereas cordycepin treatment reversed this effect, indicating that cordycepin elevated the mitochondrial membrane potential (Figure 3B). To further evaluate mitochondrial function, mitochondrial respiration was determined in H9c2 cardiomyocytes. The representative mitochondrial respiration parameters indicated that SI/R treatment dramatically decreased all of the mitochondrial respiration parameters, including basal respiration, maximal respiration, mitochondrial ATP production and spare respiratory capacity in HGHF-cultured cardiomyocytes. However, cordycepin treatment significantly increased all of these parameters as shown in Figure 3C, indicating that cordycepin treatment preserved mitochondrial function on SI/R-treated H9c2 cells. Meanwhile, mitochondrial ATP levels were also evaluated in primary cardiomyocytes. As shown in Figure 3D, cordycepin treatment enhanced mitochondrial ATP production in HG/HF-cultured SI/R cardiomyocytes. All these results demonstrated that cordycepin enhanced mitochondrial fusion and improved mitochondrial function in HG/HF-cultured SI/R cardiomyocytes.

Excess levels of mitochondrial reactive oxygen species (mtROS) are a common feature of mitochondrial dysfunction, and are thought to accelerate myocardial injury. In the present study, we found that there was a significant increase of mtROS production and a decrease of mitochondrial glutathione content in HG/HF-cultured cardiomyocytes after SI/R injury. In contrast, treatment with cordycepin significantly inhibited SI/R-induced oxidative stress and mitochondrial dysfunction (Figures 3E,F). In addition, the cardiac amount of γ-H2A.X is considered as one of the most well-known DNA damage markers of oxidative stress injury. Therefore, we further detected γH2A.X and H2A.X by Western blotting in different groups. The γ-H2A.X/H2A.X ratio was significantly elevated in SI/R condition while cordycepin treatment decreased γ-H2A.X/H2A.X ratio in HG/HF-cultured SI/R cardiomyocytes (Figure 3G). These data further illustrated that cordycepin inhibited mitochondrial ROS production and improved mitochondrial function in HG/HF-cultured SI/R cardiomyocytes.

### Cordycepin Reduced Mitochondria-dependent Intrinsic Apoptosis in HG/HF Cultured SI/R Cardiomyocytes

**FIGURE 4:**
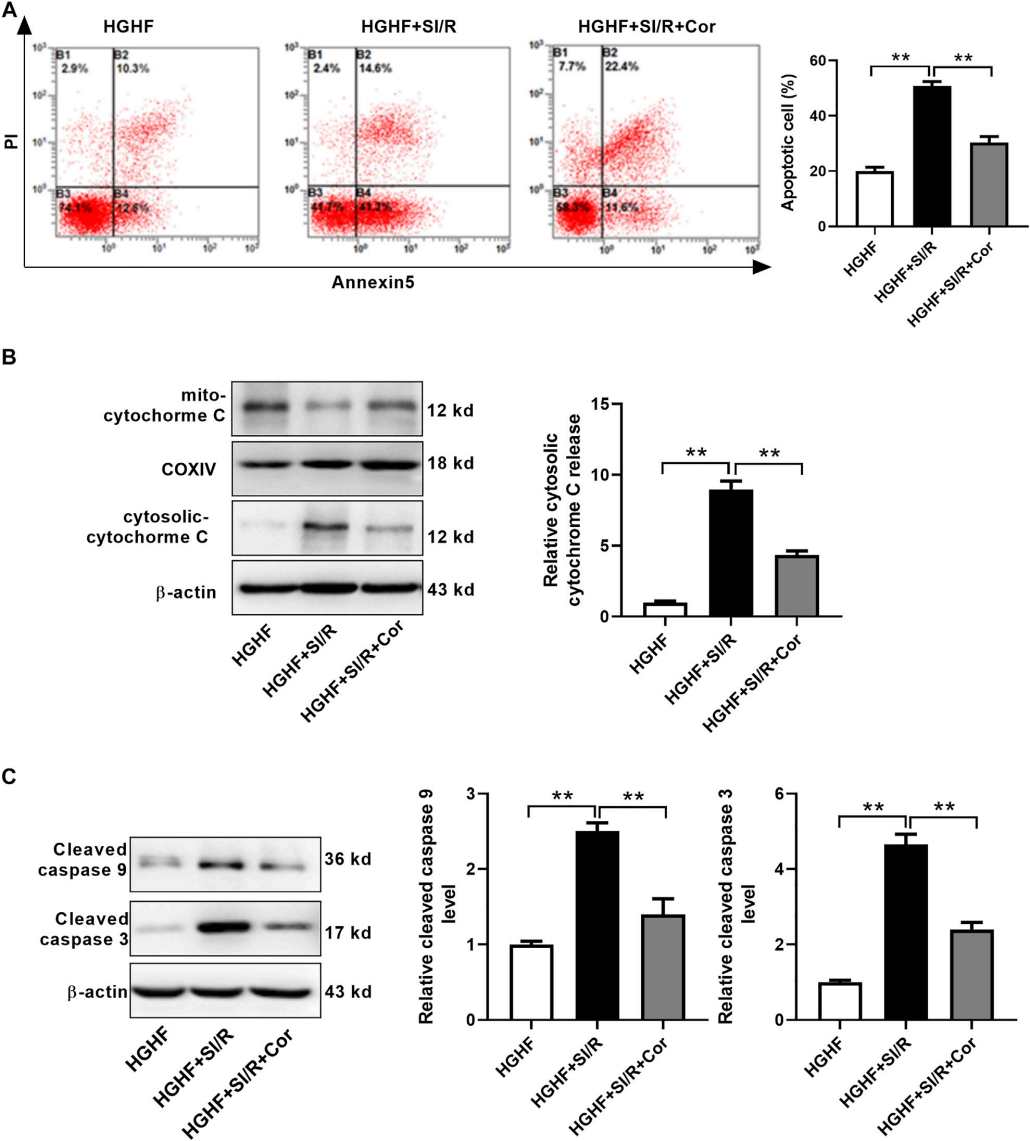
Cordycepin reduced mitochondria-dependent intrinsic apoptosis in HG/HF cultured SI/R cardiomyocytes. **(A)** Flow cytometry of apoptosis by annexin V and propidium iodide (PI) double staining (left) and quantification of apoptotic cells (right) in cardiomyocytes with treatment as indicated. **(B, C)** Western blotting and quantitative analysis of cytosolic cytochrome C release, cleaved caspase 9 levels and cleaved caspase 3 levels in cardiomyocytes. HG/HF, high-glucose/high-fat; SI/R, simulated ischemia/reperfusion; Cor, cordycepin. **p < 0.01. *n* = 3–4 wells.

To find more exact evidence that cordycepin reduces mitochondria-dependent intrinsic apoptosis, we used fluorescence-activated cell sorting (FACS) to measure cell apoptosis. As shown in Figure 4A, we found that the apoptotic rate in HG/HF cultured cardiomyocytes was greatly increased after SI/R injury, whereas cordycepin effectively prevented SI/R-induced myocardial apoptosis (*p* < 0.01). Meanwhile, cordycepin treatment significantly reduced the release of mitochondrial cytochrome c into the cytosol of SI/R cardiomyocytes (Figure 4B, *p* < 0.01). Moreover, Western blotting results revealed that cleaved caspase 9 and cleaved caspase 3 levels were both upregulated after SI/R, which was reversed by cordycepin treatment (Figure 4C, *p* < 0.01). Collectively, these findings indicate that cordycepin acts as a regulator of mitochondria-dependent intrinsic apoptosis in HG/HF-cultured SI/R cardiomyocytes.

### Knockout of Mfn2 Blocked the Cardio-Protection of Cordycepin in Diabetic Hearts

**FIGURE 5:**
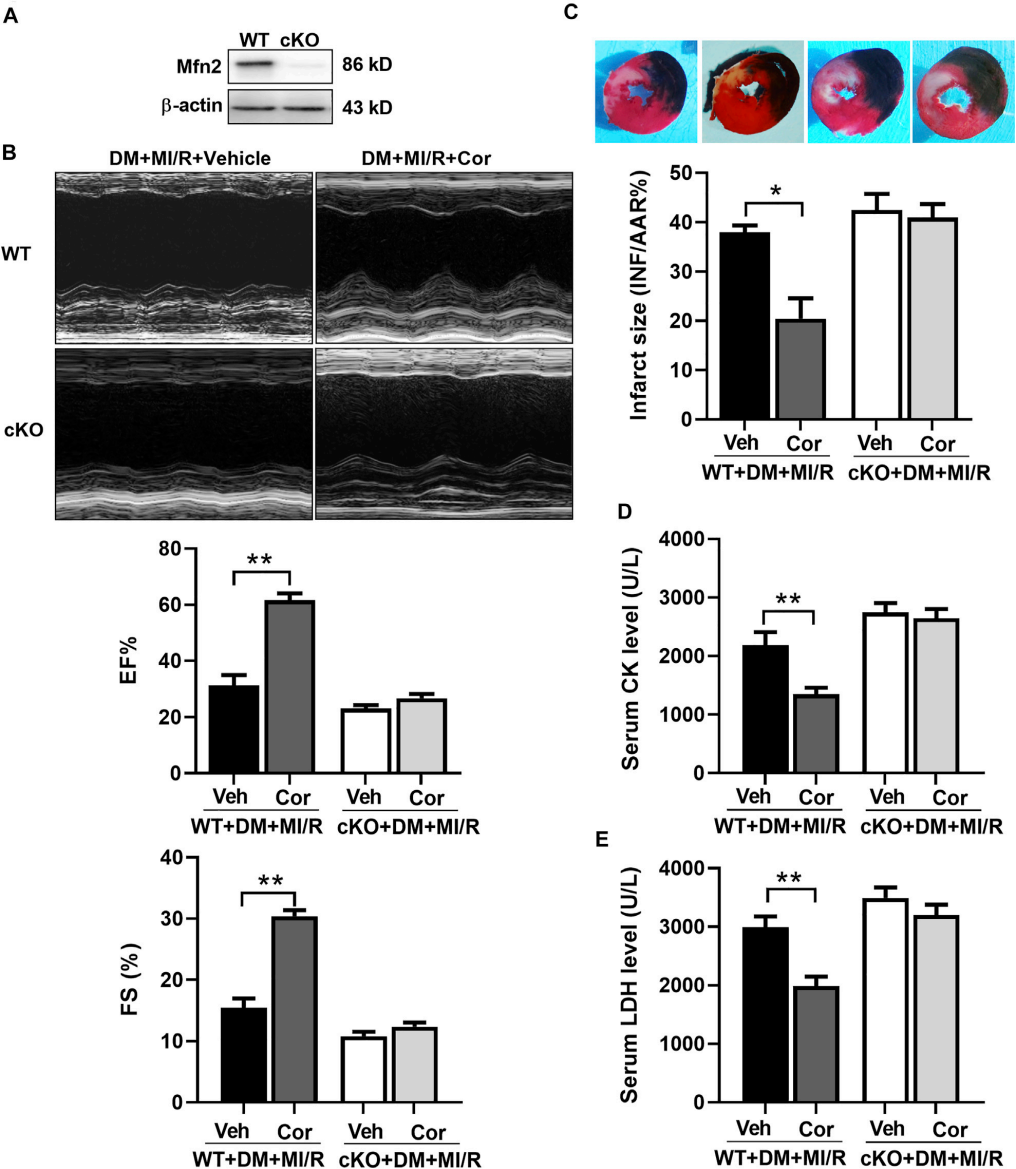
Inhibition of Mfn2 blocked the cardio-protection of cordycepin in diabetic hearts. **(A)** Western blotting of Mfn2 expression levels in mouse heart. **(B)** Left: representative echocardiograms images of diabetic mice hearts under different treatment; Right: quantitative assessment of the left ventricular ejection fraction (EF) and left ventricular fractional shortening (FS). **(C)** Top: representative photographs of heart sections. Blue-stained portion indicates nonischemic, normal region; red-stained portion, ischemic/reperfused but not infarcted region; and negative-stained portion, ischemic/reperfused infarcted region; Bottom: myocardial infarct size (INF) analysis in different groups evaluated as percentage of area at risk (AAR). **(D, E)** Serum lactate dehydrogenase (LDH) and creatine kinase (CK) level after myocardial ischemia/reperfusion injury in different groups. WT, wild type; cKO, cardiac-specific Mfn2 knockout mice; Veh, vehicle treated groups; DM, diabetes mellitus; MI/R, myocardial ischemia/reperfusion; Cor, cordycepin. *p < 0.05, **p < 0.01. *n* = 4–6 mice.

To further identify the role of Mfn2 in cordycepin-afforded cardioprotection, cardiac-specific Mfn2 knockout mice (cKO) were used in subsequent experiments. As shown in Figure 5A, Western blotting results verified the deletion of cardiac Mfn2 expression. Consistently, cardiac function was improved after cordycepin treatment in WT MI/R hearts, as evidenced by a significant increase in the left ventricular ejection fraction (EF) and left ventricular fractional shortening (FS) (Figure 5B, *p* < 0.01). The myocardial infarct size was also significantly alleviated by cordycepin treatment in WT mice (Figure 5C, *p* < 0.05), and serum CK and LDH levels were significantly decreased by cordycepin treatment in WT mice (Figures 5D,E, *p* < 0.01). However, no significant differences in cardiac function and myocardial injury were observed in Mfn2-cKO mice with or without cordycepin treatment, indicating that cordycepin induced cardioprotection in an Mfn2 dependent manner. Therefore, our results suggest that inhibition of Mfn2 blocked the cardioprotective effects of cordycepin in diabetic hearts.

### Cordycepin Increased Mfn2 Expression by AMPK Pathway

**FIGURE 6:**
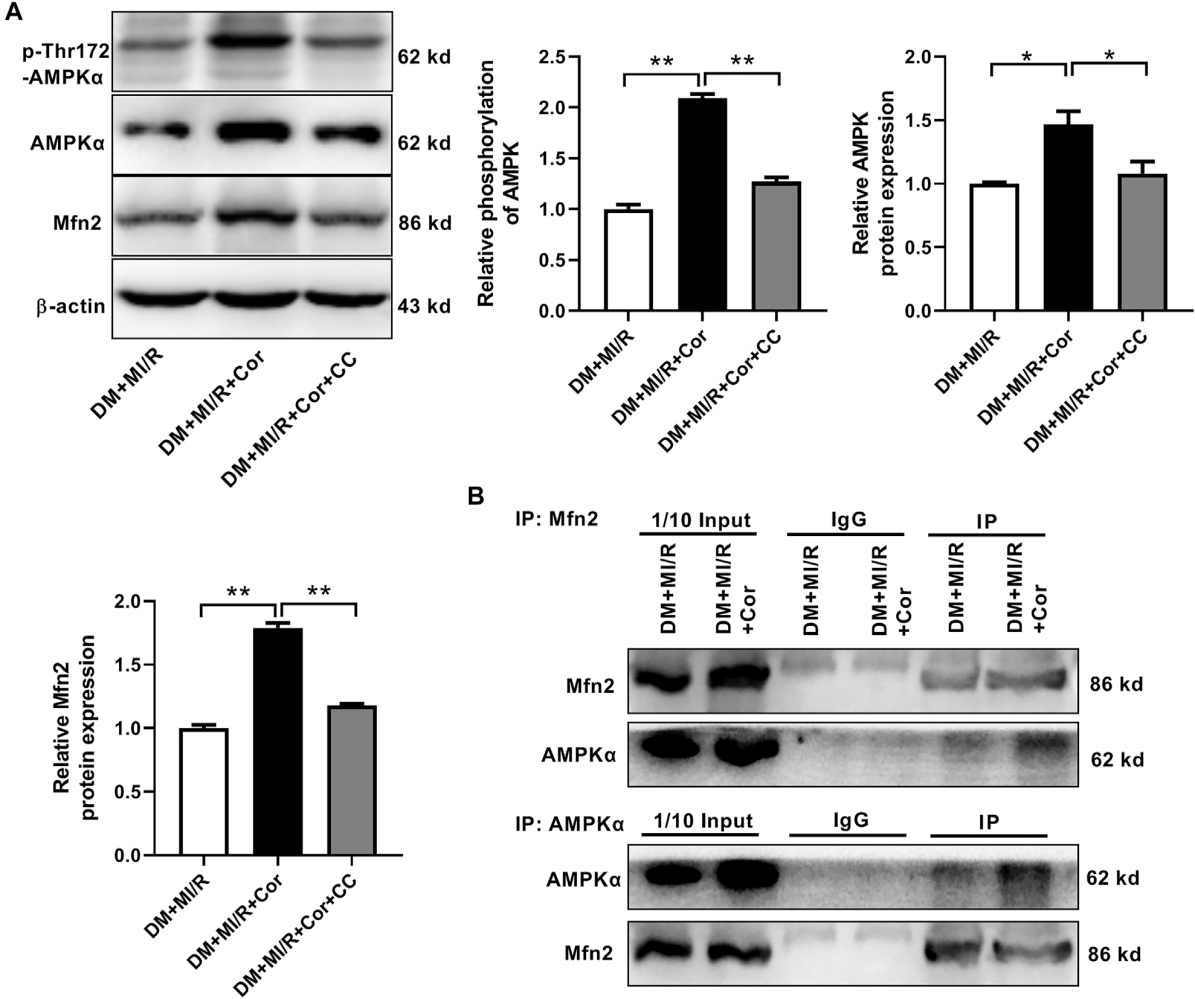
Cordycepin increased Mfn2 expression by AMPK pathway. **(A)** Western blotting and quantitative analysis of phosphorylation of AMPK, total AMPK and Mfn2 expression in mouse heart. **(B)** Co-IP of AMPK and Mfn2 in DM + MI/R mice with or without Cordycepin treatment. DM, diabetes mellitus; MI/R, myocardial ischemia/reperfusion; Cor, cordycepin; CC, compound C. *p < 0.05, **p < 0.01. *n* = 4 mice.

Next, we investigated the underlying mechanisms of cordycepin-induced Mfn2 expression. Previous studies reported that cordycepin could be converted into the AMP analog cordycepin monophosphate and subsequently activated AMPK (Hawley et al., 2020). Therefore, the phosphorylation and expression of AMPK were evaluated. As shown in Figure 6A, chronic cordycepin treatment significantly increased both the phosphorylation and the expression of AMPK, whereas pretreatment with compound C (CC), an AMPK inhibitor, completely blocked AMPK phosphorylation and expression induced by cordycepin. Interestingly, CC also inhibited cordycepin-induced Mfn2 expression, indicating that AMPK might play a critical role in Mfn2 expression. To further explore the interaction between Mfn2 and AMPK in ischemia/reperfusion injury in diabetic hearts, Co-immunoprecipitation (Co-IP) assays were performed in DM + MI/R mice. As shown in Figure 6B, an interaction between Mfn2 and AMPKα was detected, which was obviously enhanced upon cordycepin treatment. These results suggest that cordycepin increases Mfn2 expression *via* the AMPK pathway in response to MI/R injury in diabetic hearts.

## Discussion

Important findings were obtained in the present study. First, an *in vivo* MI/R model of experimental diabetic mice was used to illustrate that cordycepin treatment prevented I/R injury in diabetic hearts. Second, cordycepin treatment increased mitochondrial fusion, improved mitochondrial function, and reduced mitochondria-dependent intrinsic apoptosis. Third, knockout of Mfn2 blocked the cardioprotective function of cordycepin. Finally, the upregulation of Mfn2 in cardiomyocytes was induced by cordycepin in an AMPK-dependent manner. Collectively, our study demonstrated that cordycepin could be a potential cardioprotective agent for myocardial I/R injury in diabetes, and established a novel mechanism by which upregulated AMPK/Mfn2-dependent mitochondrial fusion contributes to the cardioprotective role of cordycepin.

It is widely acknowledged that recanalization to the ischemic myocardium can lead to various myocardial injury reactions, such as cardiomyocyte apoptosis (Frank et al., 2012). Studies have highlighted that excessive oxidative stress and inflammatory reactions aggravate cardiomyocyte apoptosis (Qiu et al., 2017). Cordycepin was isolated from Cordyceps sinensis, a commonly prescribed Chinese medicinal herb, which has been shown to be effective in anti-inflammation and anti-oxidation in a previous study (Lei et al., 2018; Han et al., 2020). In recent years, the protective role of cordycepin in I/R injury has been discovered, but most studies have focused on renal, testicular vessels, and cerebrovascular disease (Cheng et al., 2011; Okur et al., 2018; Han et al., 2020). Moreover, Park et al. first reported the protective effect of cordycepin on myocardial I/R injury in rat hearts (Park et al., 2014). However, whether cordycepin has protective effects on diabetic hearts remains unknown. In the present study, we discovered that cordycepin protects diabetic hearts from MI/R injury, indicating that cordycepin may act as a prophylactic agent against MI/R injury in patients with metabolic diseases.

Previous studies have found that the deregulation of mitochondrial dynamics may lead to metabolic dysfunction and disease (Wai and Langer, 2016). For instance, it has been reported that downregulation of Mfn2 imbalances mitochondrial dynamics, contributing to the progression of diabetic cardiomyopathy (Hu et al., 2019). In addition, of the many theories regarding the development of MI/R injury, the excessive mitochondrial fragmentation in the cardiomyocyte during the MI/R injury is an appealing one that is supported by a large foundation of experimental evidence (Lee and Yoon, 2016; Mui and Zhang, 2021). Previous studies have indicated that Mfn2, a crucial molecule that governs mitochondrial fusion and balances mitochondrial dynamics, exerts a protective role against I/R injury (Hernandez-Resendiz et al., 2020). Therefore, inhibition of excessive mitochondrial fragmentation may improve mitochondrial function and exert protective effects against MI/R injury during metabolic diseases. In the present study, we found that treatment with cordycepin directly reduced mitochondrial fragmentation and enhanced Mfn2 expression in diabetic hearts following I/R. Our *in vitro* experiments also showed that cordycepin enhanced Mfn2-medicated mitochondrial fusion and improved mitochondrial function, as evidenced by increased ATP production and elevated mitochondrial membrane potential. Furthermore, we performed experiments on the effects of cordycepin on mitochondria-dependent intrinsic apoptosis *in vitro* part of our experiments. Our results showed that cordycepin inhibits the release of mitochondrial cytochrome c into the cytosol and decreases the cleavage of caspase-9 and caspase-3 in HGHF-cultured SI/R cardiomyocytes, indicating that cordycepin inhibited the mitochondrial-mediated intrinsic apoptosis pathway in cardiomyocytes. Most importantly, inhibition of Mfn2 significantly blocked the cardioprotection of cordycepin in diabetic hearts, indicating that cordycepin induced cardioprotection, at least in part, in an Mfn2 dependent manner. However, we still cannot exclude the possibility that mitochondrial fission is involved in this process. Since mitochondrial fission regulators, such as Drp1 and Fis1, were not investigated in the present work, further studies are still needed. In contrast, cordycepin have shown to inhibit mitochondrial function by reducing Mfn2 expression and inducing mitochondrial fragmentation to suppress metastasis and migration of ovarian carcinoma cells (Wang et al., 2017). Therefore, we suspect that the regulation of Mfn2 by cordycepin is indirect, and the high mutation background of tumor cells may somehow change this regulatory relationship. In addition, different dose of cordycepin may also result in different results, hence the effect of cordycepin on mitochondrial function and dynamics in diabetic I/R hearts requires further study.

AMPK is a key regulator of cellular energy balance and cellular stress in eukaryotic cells. Previous studies have implicated that the activation of AMPK has many beneficial metabolic effects. For example, AMPK controls cellular lipid metabolism through the direct phosphorylation of Acetyl-CoA carboxylase (Garcia and Shaw, 2017). In addition, AMPK alleviates ischemic injury by regulating autophagy and inflammation in ischemic stroke (Mo et al., 2020). These results indicated a detrimental role of AMPK in cell protection. An experimental study found that cordycepin was able to activate AMPK in adipocytes (Qi et al., 2019). However, the effect of cordycepin on myocardial AMPK has not yet been explored. Our data revealed that cordycepin treatment markedly increased AMPK phosphorylation and expression, whereas inhibition of AMPK with CC significantly blocked cordycepin-increased Mfn2 expression, indicating that AMPK might play a critical role in cordycepin-mediated increase in Mfn2 expression in diabetic MI/R mice. A recent study has demonstrated that AMPK interacts with and phosphorylates Mfn2 during the mitochondrial energy crisis, suggesting that AMPK could be an upstream regulator of Mfn2 (Hu et al., 2020).

## Conclusion

**FIGURE 7:**
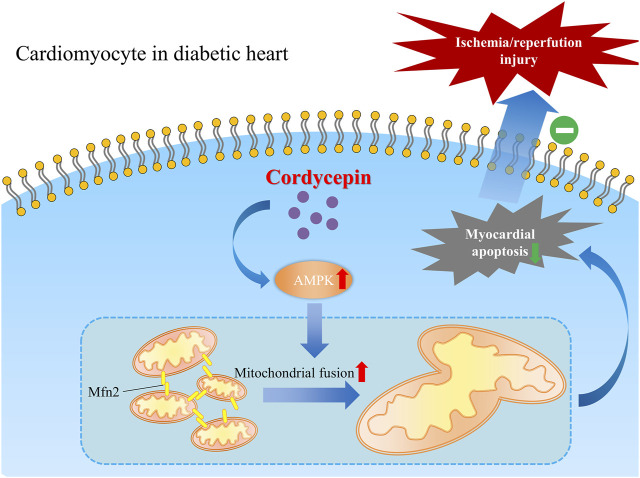
Schematic graph depicting the cardio-protective role of cordycepin during myocardial ischemia/reperfusion injury in diabetic hearts. Briefly, AMPK is upregulated by cordycepin in cardiomyocyte and plays a role in enhancement of Mfn2-mediated mitochondrial fusion and reduced myocardial apoptosis which protects diabetic heart from myocardial ischemia/reperfusion injury.

Taken together, our present study revealed a favorable role of cordycepin in protecting the diabetic heart from MI/R injury in diabetic hearts, indicating that cordycepin could be a promising effective complementary and alternative medicine for the treatment of cardiovascular diseases in diabetic patients. We also elucidated one of the possible mechanisms underlying the cardioprotective role of cordycepin in the diabetic heart *via* the AMPK/Mfn2-dependent mitochondrial fusion pathway Figure 7.

## Data Availability

The original contributions presented in the study are included in the article/Supplementary Material, further inquiries can be directed to the corresponding authors.
