# Supplementary material for: Cordycepin Decreases Ischemia/Reperfusion Injury in Diabetic Hearts via Upregulating AMPK/Mfn2-dependent Mitochondrial Fusion
Source: Front Pharmacol. 2021 Oct 20;12:754005. doi: 10.3389/fphar.2021.754005 (PMC8563605; doi:10.3389/fphar.2021.754005)
Supplement: Supplementary file 1 [file DataSheet1.pdf]

Supplementary Material

Supplementary Figures

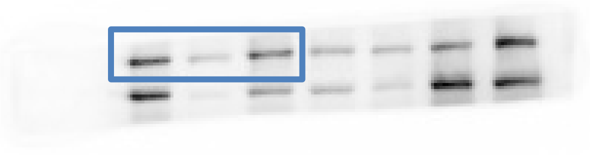

Supplementary Figure 1: Full uncropped images of western blots shown in Figure 2C-1

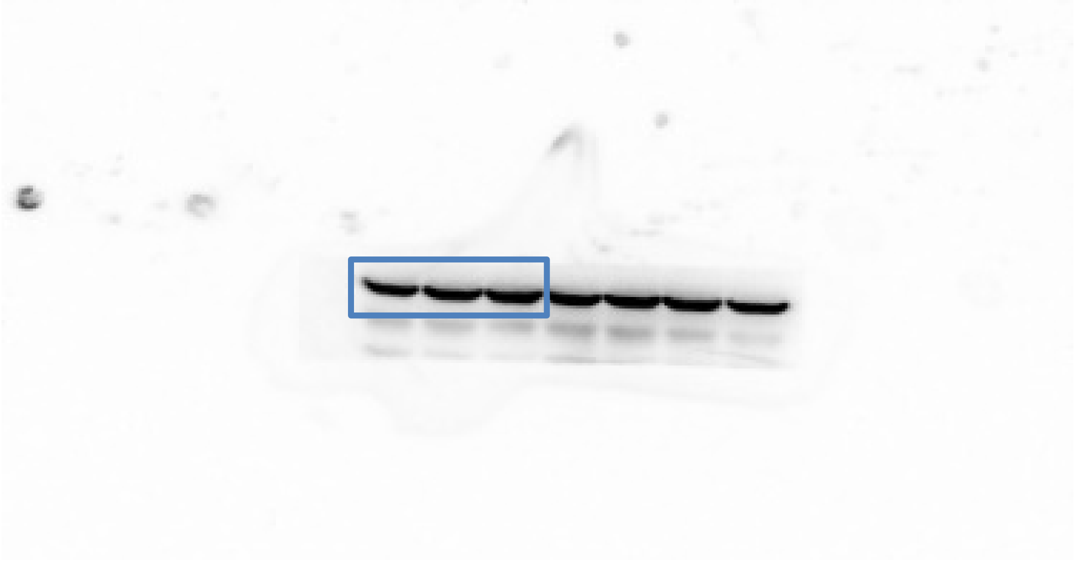

Supplementary Figure 2: Full uncropped images of western blots shown in Figure 2C-2

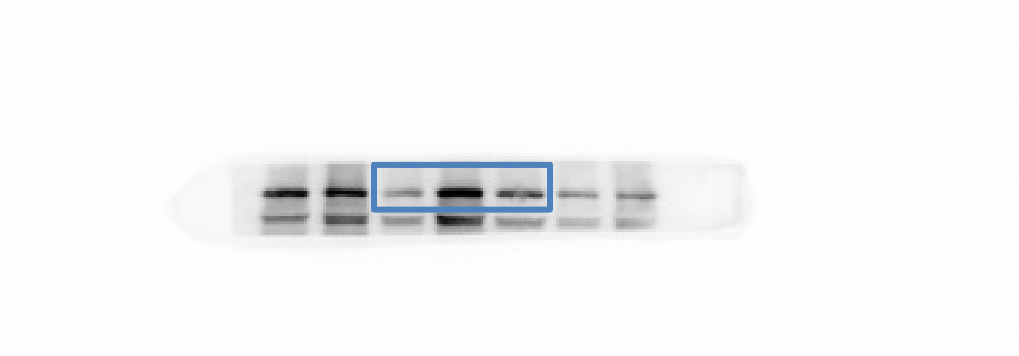

Supplementary Figure 3: Full uncropped images of western blots shown in Figure 3G-1

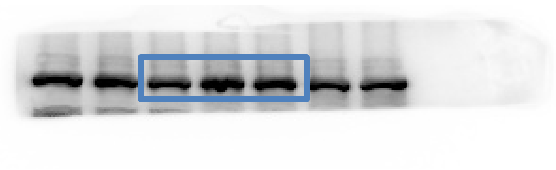

Supplementary Figure 4: Full uncropped images of western blots shown in Figure 3G-2

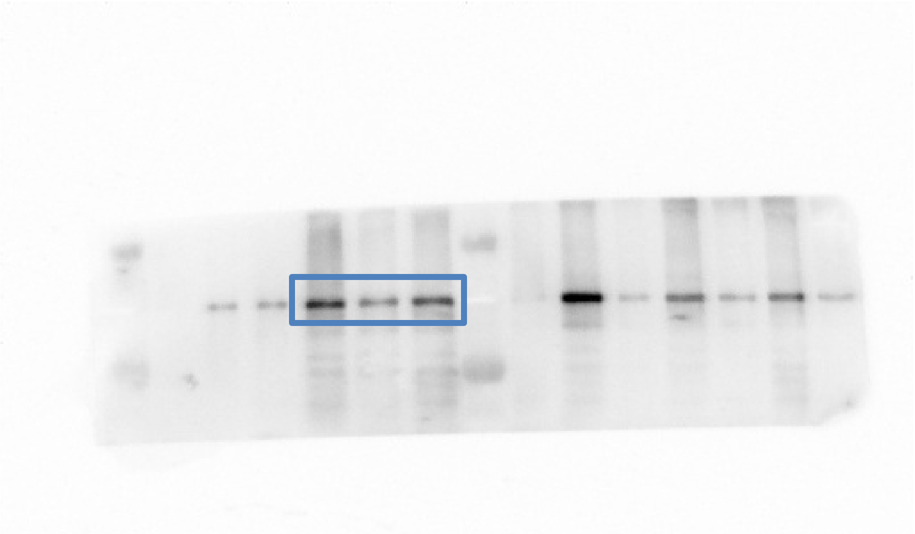

Supplementary Figure 5: Full uncropped images of western blots shown in Figure 4B-1

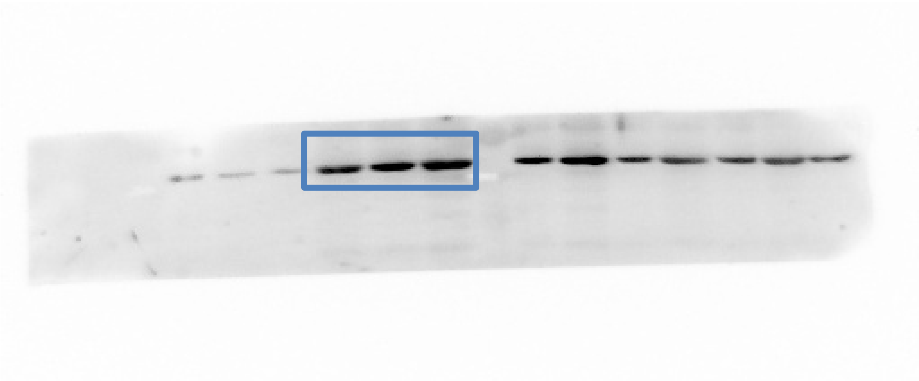

Supplementary Figure 6: Full uncropped images of western blots shown in Figure 4B-2

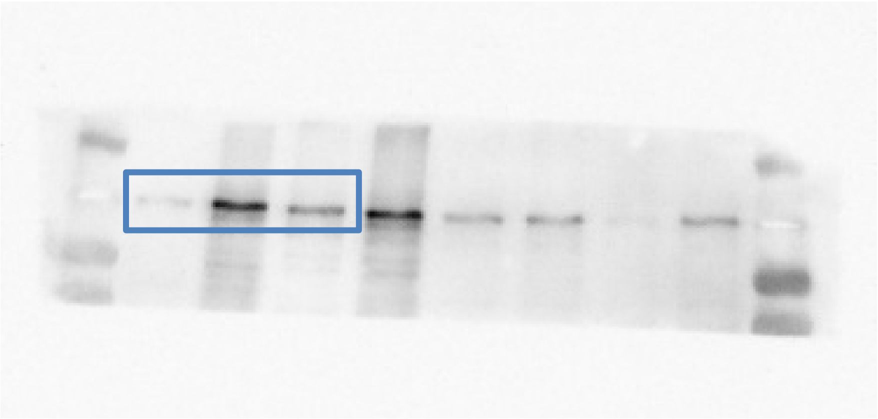

Supplementary Figure 7: Full uncropped images of western blots shown in Figure 4B-3

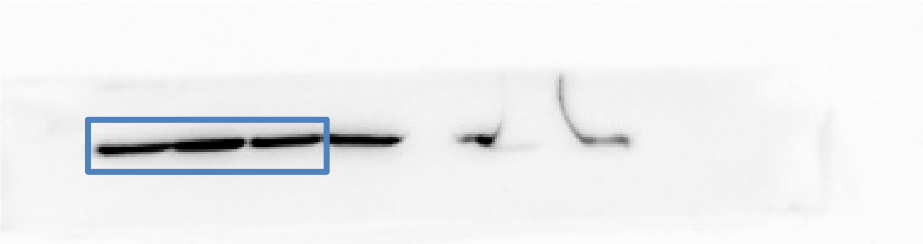

Supplementary Figure 8: Full uncropped images of western blots shown in Figure 4B-4

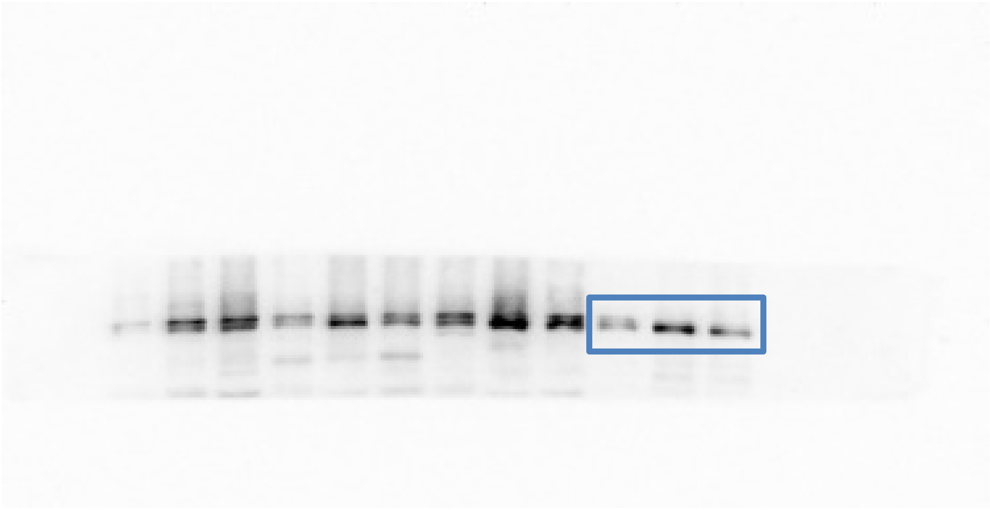

Supplementary Figure 9: Full uncropped images of western blots shown in Figure 4C-1

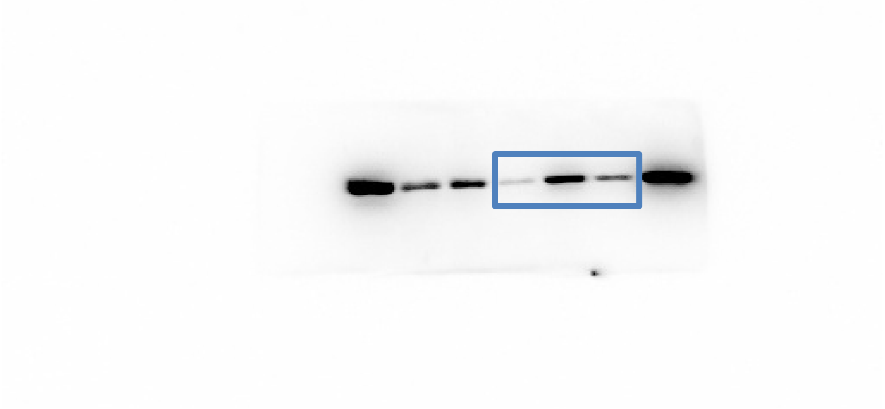

Supplementary Figure 10: Full uncropped images of western blots shown in Figure 4C-2

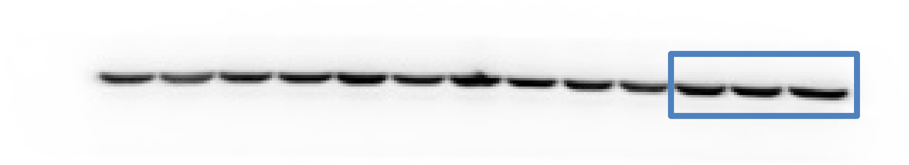

Supplementary Figure 11: Full uncropped images of western blots shown in Figure 4C-3

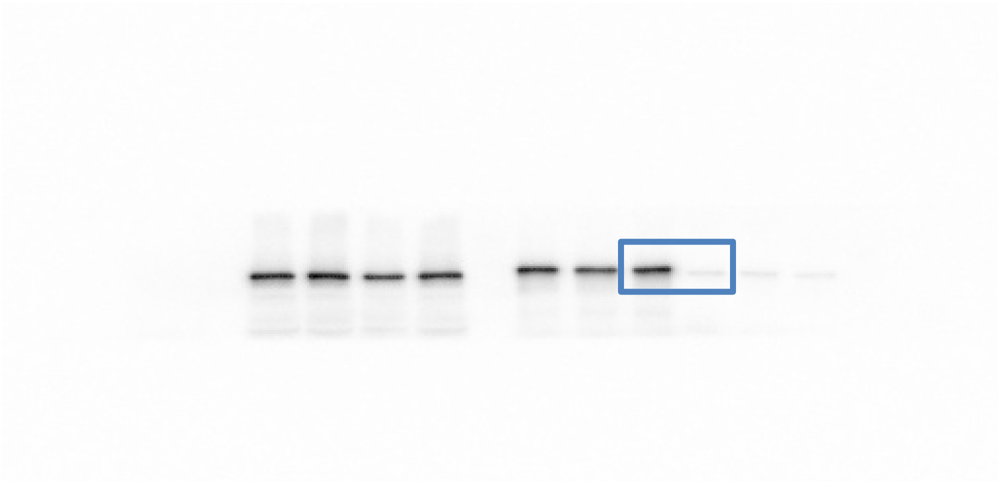

Supplementary Figure 12: Full uncropped images of western blots shown in Figure 5A-1

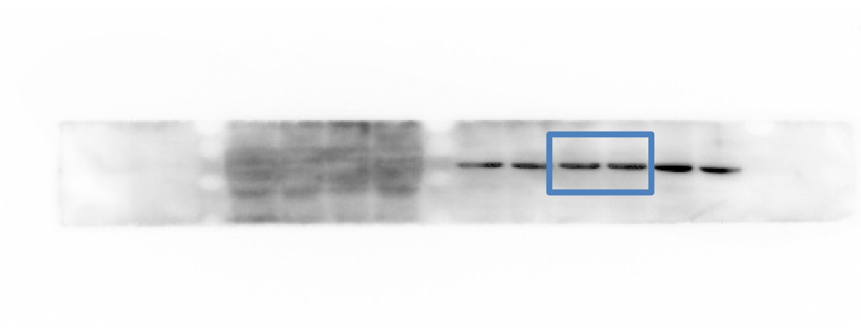

Supplementary Figure 13: Full uncropped images of western blots shown in Figure 5A-2

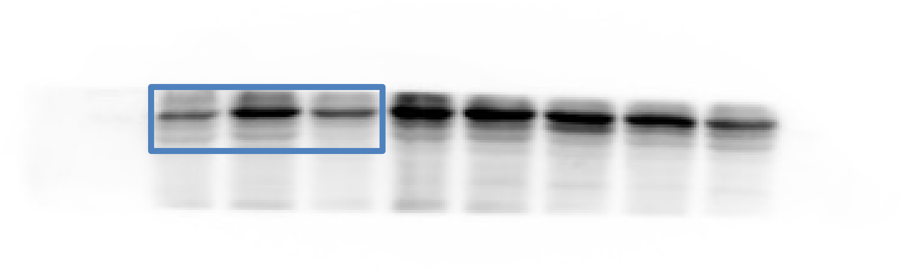

Supplementary Figure 14: Full uncropped images of western blots shown in Figure 6A-1

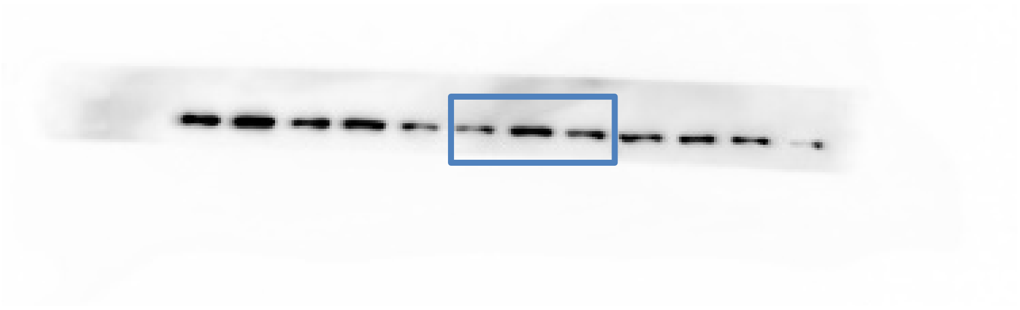

Supplementary Figure 15: Full uncropped images of western blots shown in Figure 6A-2

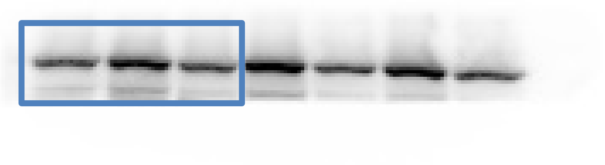

Supplementary Figure 16: Full uncropped images of western blots shown in Figure 6A-3

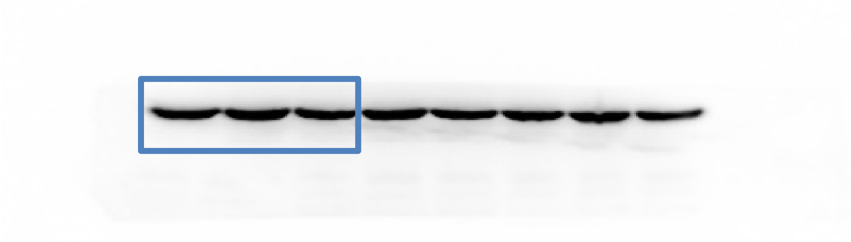

Supplementary Figure 17: Full uncropped images of western blots shown in Figure 6A-4

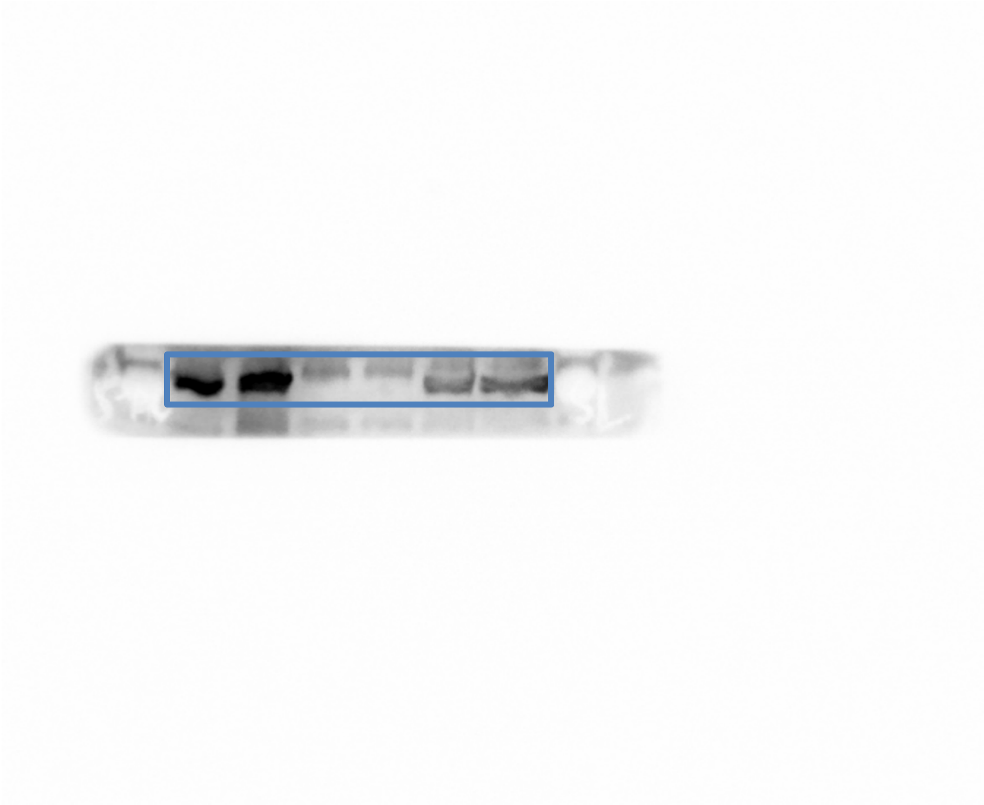

Supplementary Figure 18: Full uncropped images of western blots shown in Figure 6B-1

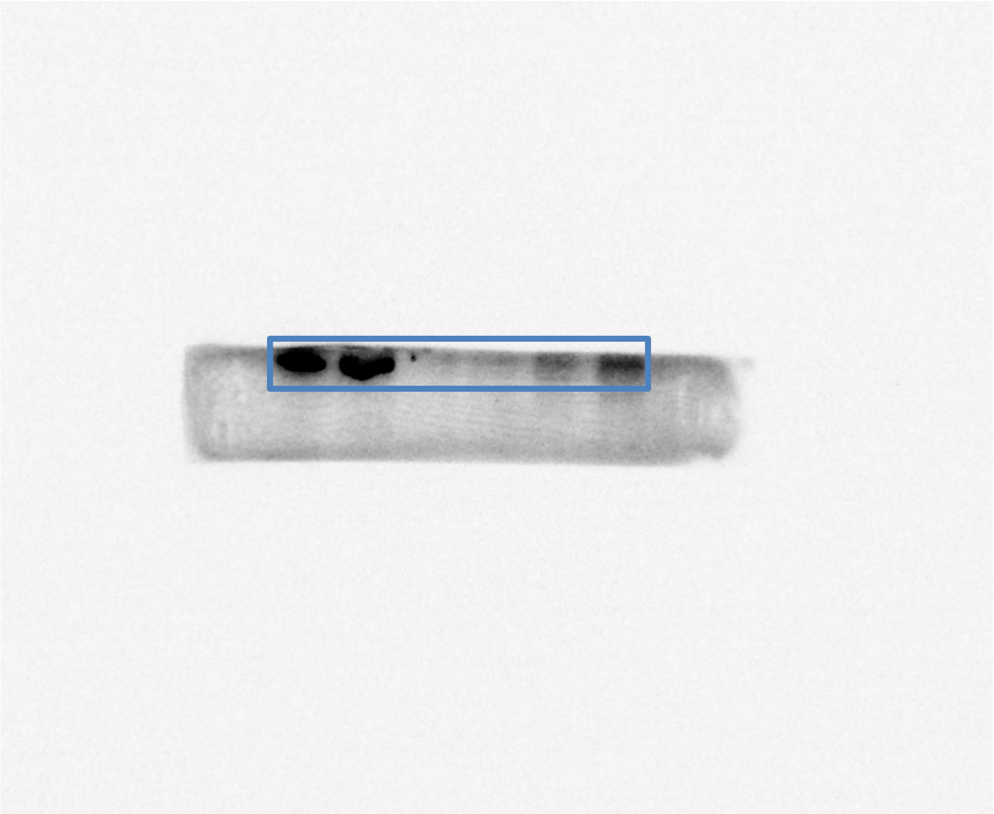

Supplementary Figure 19: Full uncropped images of western blots shown in Figure 6B-2

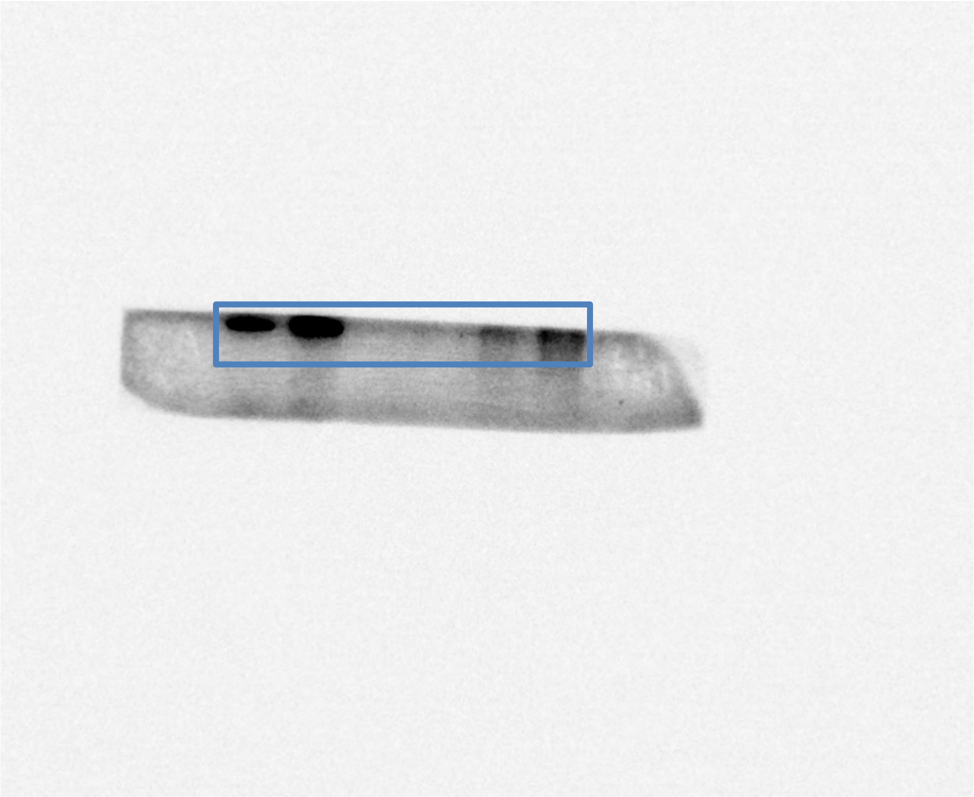

Supplementary Figure 20: Full uncropped images of western blots shown in Figure 6B-3

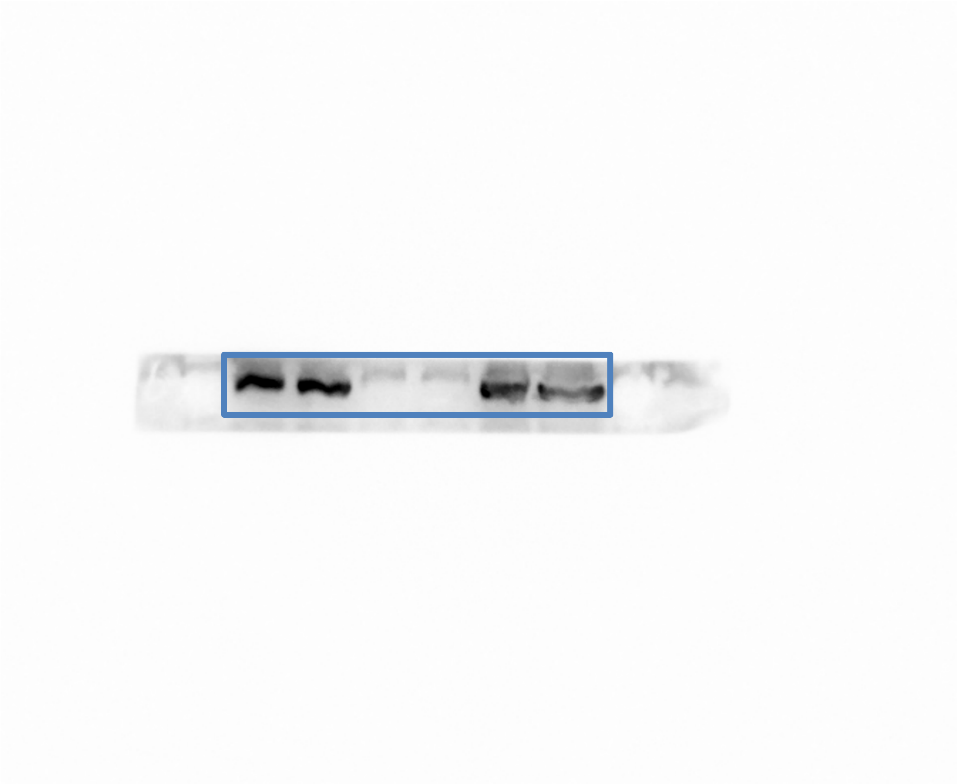

Supplementary Figure 21: Full uncropped images of western blots shown in Figure 6B-4
